# Understanding the molecular mechanisms of anti-trafficking therapies and their clinical relevance in inflammatory bowel disease

**DOI:** 10.1016/j.mucimm.2023.08.001

**Published:** 2023-09-03

**Authors:** Saurabh Mehandru, Jean-Frederic Colombel, Julius Juarez, James Bugni, James O. Lindsay

**Affiliations:** 1 The Henry D. Janowitz Division of Gastroenterology, Icahn School of Medicine at Mount Sinai, New York, NY, USA; 2 Takeda Pharmaceuticals U.S.A., Inc., Lexington, MA, USA; 3 Blizard Institute, Barts and The London School of Medicine and Dentistry, London, UK; 4 Department of Gastroenterology, Royal London Hospital, Barts Health NHS Trust, London, UK

## Abstract

In patients with inflammatory bowel disease (IBD), a combination of dysbiosis, increased intestinal permeability, and insufficient regulatory responses facilitate the development of chronic inflammation, which is driven by a complex interplay between the mucosal immune system and the environment and sustained by immune priming and ongoing cellular recruitment to the gut. The localization of immune cells is mediated by their expression of chemokine receptors and integrins, which bind to chemokines and adhesion molecules, respectively. In this article, we review the mechanisms of action of anti-trafficking therapies for IBD and consider clinical observations in the context of the different mechanisms of action. Furthermore, we discuss the evolution of molecular resistance to anti-cytokines, in which the composition of immune cells in the gut changes in response to treatment, and the potential implications of this for treatment sequencing. Lastly, we discuss the relevance of mechanism of action to combination therapy for IBD.

## INFLAMMATORY BOWEL DISEASE (IBD) PATHOPHYSIOLOGY

IBD is a chronic inflammatory disorder that is placing a growing burden on healthcare systems^1,2^. The incidence of IBD in Western countries is 12–26 per 100,000 people per year and is rapidly increasing in newly industrialized countries in Asia and Latin America^2^. The major forms of IBD include Crohn’s disease (CD) and ulcerative colitis (UC)^1^. CD is characterized by patchy, transmural inflammation that can manifest anywhere in the gastrointestinal tract, whereas UC is limited to the colonic mucosa and submucosa^1^. CD and UC are progressive diseases, with chronic inflammation driving the development of complications such as fibrosis, stenosis, abscesses, and colitis-associated colon cancer^1,3^.

Although the precise etiology of IBD is unknown, it involves multiple elements, such as genetic susceptibility, environmental factors (including diet), and the composition of the intestinal microbiome^1^. The complex interplay among these factors disturbs gut homeostasis, impairs intestinal barrier function, and allows the translocation of commensal microbes and luminal antigens into the immunologically rich lamina propria. Indeed, decreased microbial diversity and increased intestinal permeability are reported to precede the development of IBD^4^. Upon exposure to the luminal contents, innate and adaptive immune cells initiate an inflammatory cascade that leads to the production of cytokines and chemokines involved in immune cell recruitment, differentiation, and activation. Trafficking of immune cells to the gut amplifies the local immune response and drives intestinal inflammation. In healthy hosts, this inflammatory immune response is self-limiting and is resolved by regulatory responses once potential pathogens have been cleared. However, in IBD, a combination of dysbiosis, increased intestinal permeability, and insufficient regulatory responses facilitate the development of chronic inflammation, which is sustained by enhanced immune cell priming and the ongoing recruitment of immune cells to the gut^5^.

## STEADY-STATE IMMUNE CELL TRAFFICKING

The controlled trafficking of immune cells into the intestinal mucosa plays an essential role in gut homeostasis and immunosurveillance^6,7^. Immune cell migration into peripheral tissues is a multi-step process that begins with rolling adhesion when selectins on the surface of immune cells bind reversibly to their corresponding ligands on the endothelium^6^. These interactions cannot anchor cells against the shearing force of blood flow; therefore, cells appear to roll along the endothelium. Rolling adhesion facilitates the formation of tight binding by exposing immune cells to tissue-specific mediators, such as chemokines, and supporting integrin-dependent arrest. Chemokines secreted by tissue-resident cells bind to specific receptors on the immune cell, triggering a conformational change of integrins. This stabilizes the interactions among integrins on the surface of immune cells and adhesion molecules expressed by endothelial cells, eventually resulting in firm adhesion and extravasation, when the immune cell traverses the endothelial barrier to enter the sub-endothelial tissue. Lastly, immune cells migrate through the tissue under the influence of chemokine or other chemoattractant gradients. The expression profile of chemokine receptors, integrins, and their ligands forms a “zip code” that directs the localization of immune cells^6^.

### Gut-associated lymphoid tissue trafficking

In the intestine, secondary lymphoid organs comprise the draining mesenteric lymph nodes and the gut-associated lymphoid tissue (GALT). The GALT represents key antigen sampling and adaptive immune priming sites in the intestinal wall and includes Peyer’s patches and isolated lymphoid follicles^8^. Peyer’s patches are found throughout the small intestine, with the greatest density in the terminal ileum, and are overlaid with a follicle-associated epithelium rich in specialized antigensampling epithelial cells known as microfold cells (or M cells), which use a process known as transcytosis to sample and transport luminal antigens to the sub-epithelium, where they can be presented to adaptive immune cells^8^.

**Fig. 1:**
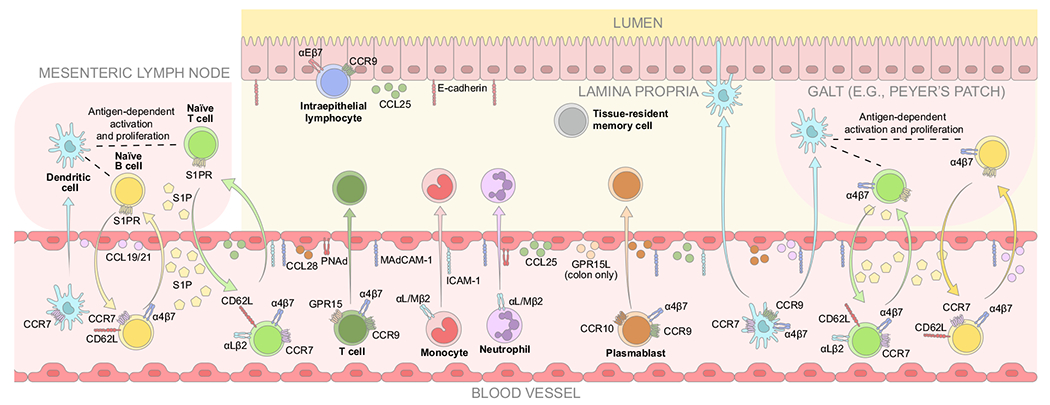
Steady-state immune cell trafficking. CCL = C-C chemokine ligand; CCR = C-C chemokine receptor; CD62L = cluster of differentiation 62 ligand (L-selectin); GPR = G protein-coupled receptor; GPR15L = G protein-coupled receptor 15 ligand; ICAM-1 = intracellular adhesion molecule 1; MAdCAM-1 = mucosal vascular addressin cell adhesion molecule 1; PNAd = peripheral lymph node addressin; S1P = sphingosine-1 phosphate; S1PR = sphingosine-1 phosphate receptor.

Naïve T cells express cluster of differentiation 62 ligand (CD62L; L-selectin), αLβ2 and α4β7 integrins, and C-C chemokine receptor (CCR) 7 to facilitate trafficking to the GALT^9–11^. Interactions between CD62L and peripheral lymph node addressin (PNAd) and between α4β7 integrin and mucosal addressin cell adhesion molecule 1 (MAdCAM-1) mediate tethering and rolling on high endothelial venules, and integrin activation is signaled by CCR7 interactions with its C-C chemokine ligands (CCLs) 19 and 21. Firm adhesion is achieved by the binding of αLβ2 integrin and intracellular adhesion molecule 1 (ICAM-1) and/or α4β7 integrin and MAdCAM-1 (Fig. 1)^9–11^. If a naïve T cell encounters its cognate antigen in the GALT, it undergoes differentiation and clonal expansion to form a pool of effector T (Teff) cells^6,12^. During conventional T-cell expansion, naïve T cells can differentiate into Teff cells, regulatory T (Treg) cells, central memory (Tcm) cells, effector memory cells, and tissue-resident memory (Trm) cells, which express different homing markers relating to their functions^13,14^. In the absence of antigen stimulation, naïve T cells recirculate to patrol other secondary lymphoid organs. The egress of T cells is regulated by sphingosine-1 phosphate (S1P) in the blood and lymph, which binds to S1P receptors on T cells and draws them back into the circulation^15^. In comparison to blood and lymph, levels of S1P are relatively low in lymphoid tissue, owing to S1P catabolic enzymes. Local S1P gradients between lymphoid tissues and the circulation are essential for regulating the egress of T cells from secondary lymphoid organs, including the GALT and the thymus^16^. Tcm cells express a similar homing marker profile to naïve T cells, linked with their function of recirculation throughout the secondary lymphoid organs and peripheral blood^6,11^.

B cells are also recruited to the GALT via CD62L and α4β7 integrin interactions with MAdCAM-1 on high endothelial venules, as well as activation by CCR7–CCL21 binding^6^. Like T cells, the egress of B cells from secondary lymphoid tissues is regulated by S1P gradients between lymphoid tissues and the circulation^17^.

Dendritic cells migrate from the lamina propria to the draining mesenteric lymph nodes in a process mediated by CCR7 and its ligands CCL19 and CCL21^18,19^. Dendritic cells in the mesenteric lymph nodes and GALT can activate naïve lymphocytes and induce the upregulation of α4β7 integrin and CCR9 via retinoic acid signaling^19–22^.

### Lamina propria trafficking

Upon egress from the GALT into the lymphatic drainage and eventually into the venous circulation, GALT-primed lymphocytes localize back to the intestinal lamina propria via α4β7 integrin and CCR9^23–25^. During trafficking of T cells to the small intestine, CCR9 binds to CCL25, which leads to conformational changes in α4β7 integrin that stabilize its interaction with MAdCAM-1 on venules (Fig. 1)^22,26^. In the colon, the chemokine signal enhancing the trafficking of T cells is likely facilitated by G protein-coupled receptor 15 and its ligand^26–28^. In general, B cell trafficking to the small intestine appears to be controlled in a similar way to that of T cells via CCR9 and α4β7 integrin; however, homing of plasmablasts to both the small and large intestines can be facilitated by the binding of CCR10 to its ligand CCL28^26^. α4β7 integrin and CCR9 also mediate the gut homing of innate lymphoid cells^29^. Conversely, homing of myeloid cells, such as monocytes and neutrophils, is predominantly mediated by selectins and the interaction of ICAM-1 with αLβ2 and αMβ2 integrins^6,30^. However, α4β7 integrin is expressed in eosinophils and may play a role in trafficking these cells^31,32^. Furthermore, dendritic cells traffic to the gut via CCR9 and α4β7 integrin^23–25^.

### Epithelial trafficking and residency

Trm cells are memory T cells that stably reside in nonlymphoid tissues and can generate rapid site-specific effector responses. These T cells typically have high expression of molecules such as CD69, CD103 αEβ7), and CD49a and low expression of homing markers such as S1P receptor 1, CD62L, and CCR7^33^. In the lower gastrointestinal tract, up to 90% of CD4+ and CD8+ T cells display phenotypic features of Trm cells^33^.

Gut intraepithelial lymphocytes (IELs) are a heterogeneous population of cells (including γδ T cells), consisting primarily of antigen-experienced T cells that are located within the epithelial monolayer^34–36^. There are two major subsets of IELs: natural IELs, which are activated in the thymus by self-antigens, and induced IELs, which originate from T cells activated by peripheral antigens in secondary lymphoid organs^35^. IELs migrate from the thymus (natural) or the lamina propria (induced) to the intraepithelial compartment facilitated by the interaction between CCR9 and CCL25 (Fig. 1)^22,35,37^. Additionally, binding of αEβ7 integrin on IELs to E-cadherin on the basolateral surface of enterocytes mediates their adhesion and retention in the intestinal epithelium (Fig. 1)^35,37,38^. IELs provide a first line of defense against pathogens and protect the integrity of the intestinal epithelium by preventing uncontrolled immune cell infiltration and excessive immune responses mediated by T cells^35^. However, IELs may contribute to immunopathological responses and exacerbate inflammation in IBD^35,39^.

## ABERRANT IMMUNE CELL TRAFFICKING IN IBD

**Fig. 2:**
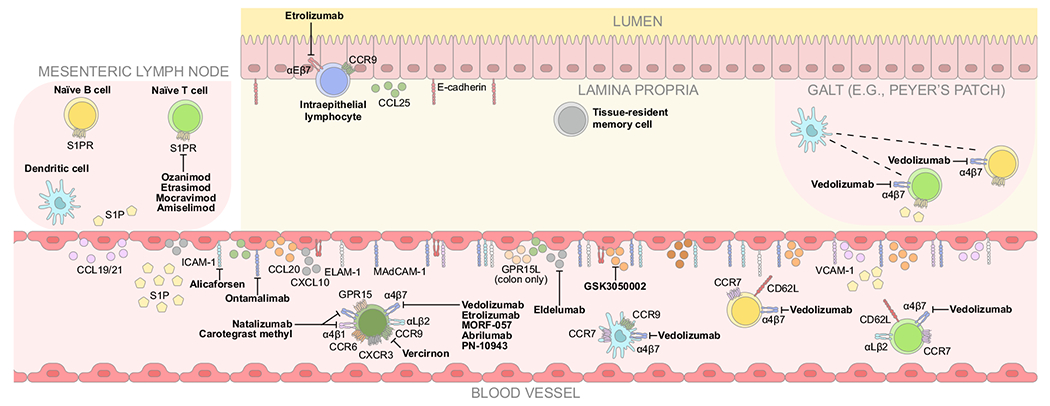
Aberrant trafficking in inflammatory bowel disease and mechanisms of action of anti-trafficking therapies. CCL = C-C chemokine ligand; CCR = C-C chemokine receptor; CD62L = cluster of differentiation 62 ligand (L-selectin); CXCL = C-X-C ligand; CXCR = C-X-C receptor; ELAM-1 = endothelial leukocyte adhesion molecule-1; GPR = G protein-coupled receptor; GPR15L = G protein-coupled receptor 15 ligand; ICAM-1 = intracellular adhesion molecule 1; MAdCAM-1 = mucosal vascular addressin cell adhesion molecule 1; PNAd = peripheral lymph node addressin; S1P = sphingosine-1 phosphate; S1PR = sphingosine-1 phosphate receptor; VCAM-1 = vascular cell adhesion molecule 1.

Increased immune cell trafficking is a key feature of the aberrant immune response in IBD^22,40^. In IBD, augmented pro-inflammatory cytokines, including anti-tumor necrosis factor α (TNFα), enhance the expression of MAdCAM-1 and increase gut-selective trafficking^9^. Expression of other cell adhesion molecules normally absent or with limited expression in the gut at homeostasis, such as endothelial leukocyte adhesion molecule-1, vascular cell adhesion molecule 1 (VCAM-1), ICAM-1, and PNAd are also induced or elevated on intestinal endothelial cells, resulting in a sustained influx of immune cells (Fig. 2)^41–43^. In contrast to MAdCAM-1, these pathways facilitate trafficking systemically and are not gut-selective^44–46^. ICAM-1 on the surface of endothelial cells tethers to β2 integrins^44^, whereas VCAM-1 binds to both α4β1 and α4β7 integrins^47,48^.

In IBD, immune cell recruitment is enhanced by increased chemokine signaling^22,26^. For example, elevated CCL2, CCL7, CCL20, CCL25, and C-X-C ligand (CXCL) 10 levels are present in patients with IBD^49–52^. The interaction between CXCL10 and its receptor C-X-C receptor (CXCR) 3 has been implicated in the migration of T helper (Th) 1 cells^53^, whereas CCL20 binding to CCR6 mediates CD4+ T-cell recruitment to the gut during inflammation (Fig. 2)^54^. Elevated expression of both adhesion molecules and chemokines is associated with increased recruitment of monocytes and the accumulation of macrophages^55^. In turn, these innate immune cells produce pro-inflammatory mediators that amplify adhesion molecule expression and chemokine signaling to promote T-cell recruitment^55^. Upon recruitment, T cells secrete pro-inflammatory cytokines that promote further recruitment and activation of other immune cells, which damage the mucosa and release additional inflammatory mediators that perpetuate chronic inflammation in the gut^40,56^.

In addition to increased trafficking of pro-inflammatory T cells, functional defects in anti-inflammatory Treg cells, as well as their ability to traffic to inflamed intestinal lesions, have also been identified in patients with IBD^57^. Treg cells support intestinal homeostasis by mediating oral tolerance to dietary antigens and suppressing pathogenic T-cell responses^58,59^.

## ANTI-TRAFFICKING THERAPIES FOR IBD

Based on their mechanism of action (MoA), advanced therapies for IBD can be categorized into anti-cytokine and anti-trafficking therapies. Anti-cytokines aim to reduce intestinal inflammation by neutralizing one or more pro-inflammatory cytokines, including TNFα (e.g. adalimumab, golimumab, infliximab, and certolizumab pegol), interleukin-12/23 (IL-12/23; e.g. ustekinumab), or IL-23 (e.g. guselkumab, mirikizumab, and risankizumab) or by inhibition of cytokine signaling pathways, such as Janus kinase inhibitors^60–62^. Anti-trafficking therapies limit the trafficking of immune cells to the gut, particularly the excessive trafficking of T cells that secrete pro-inflammatory cytokines and perpetuate the inflammatory response in IBD. Several anti-trafficking therapies are approved or in clinical development for the treatment of UC and/or CD (Table 1^63–116^; Fig. 2).

### Anti-chemokine therapies

Vercirnon is an oral small molecule antagonist of CCR9^63,64^. Binding of CCR9 and CCL25 stabilizes the interaction between α4β7 integrin and MAdCAM-1 during immune cell trafficking to the gut^103^. In the phase III SHIELD-1 trial (*n* = 608), vercirnon failed to demonstrate efficacy over placebo as an induction therapy in patients with moderately to severely active CD, and the clinical development program was subsequently terminated^64^. Eldelumab is an intravenously administered fully human monoclonal antibody (mAb) targeting CXCL10^65–67^. The interaction between CXCL10 and CXCR3 has been implicated in the migration of Th1 cells^53^. Eldelumab failed to achieve its efficacy endpoints in phase II clinical trials in patients with moderately to severely active UC and CD^65–67^. Redundancy is a potential explanation for the lack of efficacy of both vercirnon and eldelumab. Because chemokines can bind multiple receptors and chemokine receptors interact with multiple ligands, inhibiting the binding of a single chemokine or receptor may not be sufficient to inhibit immune cell trafficking if alternative chemokine signaling pathways remain intact.

GSK3050002 is an intravenously administered humanized immunoglobulin (Ig) G_1_ mAb targeting CCL20^68^. Binding of CCL20 to CCR6 is involved in the homing of CD4+ T cells to the gut during inflammation^54^. GSK3050002 was well tolerated in a phase I “first-in-human” study in healthy volunteers^68^; however, in a subsequent 26-week toxicity study in cynomolgus monkeys, a high incidence of vascular and organ inflammation was observed microscopically in several tissues, resulting in the termination of clinical development for this compound^69^. It was hypothesized that immune complexes of drug aggregates and complement accumulated over time and triggered immune complex disease^69^. There are concerns generally regarding the potential safety of anti-chemokine therapies, owing to the wide-spread expression of chemokines and chemokine receptors^104^.

### Anti-integrin therapies

Natalizumab is an intravenously administered humanized mAb targeting the α4 subunit of heterodimeric integrins α4β7 and α4β1^70^. Natalizumab inhibits α4β7 integrin and MAdCAM-1 binding to impede immune cell trafficking to the gut^71^. However, natalizumab also inhibits systemic immune cell trafficking via inhibition of α4β1 integrin binding to VCAM-1^71^. In the Efficacy of Natalizumab as Active Crohn’s Therapy ENACT-1 (n = 905) and ENACT-2 (*n* = 339) phase III clinical trials in patients with active CD, natalizumab had a similar rate of response to placebo at induction but achieved a higher rate of sustained response during maintenance^70^. Natalizumab is approved by the US Food and Drug Administration (FDA) as a treatment for moderately to severely active CD but is only available via a restricted distribution program called the CD-TOUCH Prescribing Program because of the risk of progressive multifocal leukoencephalopathy (PML)^72^, a demyelinating brain disorder caused by the John Cunningham virus^105^. The increased risk of PML is thought to be due to natalizumab blocking the interaction between α4β1 integrin and VCAM-1, which prevents the transmigration of lymphocytes across the blood–brain barrier and therefore reduces immunosurveillance in the brain^106^. Another α4 integrin inhibitor, carotegrast methyl, an orally administered small molecule, is available in Japan for patients with moderate UC who have not sufficiently responded to 5-aminosalicylic acid^73,74^.

Etrolizumab is a subcutaneously administered humanized mAb targeting the β7 subunit of the heterodimeric integrins α4β7 and αEβ7^39,107^. Etrolizumab inhibits the interaction between α4β7 integrin and MAdCAM-1, thereby inhibiting the trafficking of immune cells to the gut^39^, and blocks the binding between αEβ7 and E-cadherin, an interaction that mediates the adhesion and retention of IELs, and potentially Trm cells^108–110^. Evidence from a humanized mouse model suggests that combined α4β7 and αEβ7 integrin blockade results in a superior reduction of cytotoxic CD8+ T cells and CD4+ IL-9-secreting Th9 cells in the colon than α4β7 integrin blockade only^109^. Th9 cells and IL-9 induce inflammation and are known to exacerbate IBD; however, their roles have not been fully elucidated^111^.

Etrolizumab has been investigated in six phase III clinical trials in patients with moderately to severely active UC (HIBISCUS I and II, LAUREL, HICKORY, and GARDENIA) and moderately to severely active CD (BERGAMOT)^79^. Among patients with UC, etrolizumab was shown to be superior to placebo for the induction of remission at week 10 in HIBISCUS I (*n* = 358) but not in the identical HIBISCUS II (*n* = 358) trial^75^. In the LAUREL study (induction, *n* = 359; maintenance, *n* = 214), there was no significant difference between etrolizumab and placebo for maintenance of clinical remission at week 62 among patients with a clinical response at week 10^76^. In the HICKORY study (induction, *n* = 609; maintenance, *n* = 259), which included patients who had previously received an anti-TNFα agent, etrolizumab was superior to placebo for the induction of remission at week 14, but no significant differences were observed for remission at week 66 among patients with a clinical response at week 14^77^. In the GARDENIA study (*n* = 397), etrolizumab was not superior to infliximab for the primary endpoint of clinical response at week 10 and clinical remission at week 54^78^. Among patients with CD in the BERGAMOT study (induction, *n* = 385; maintenance, *n* = 487), the proportions of patients who achieved clinical remission and endoscopic improvement were significantly higher with etrolizumab than placebo at week 66 but not at week 14^112^. The development of etrolizumab for the treatment of UC and CD has since been terminated^80^.

The reason for the failure of etrolizumab to meet its primary endpoints in some trials is not known. Pharmacokinetic analyses demonstrated that etrolizumab reached expected drug exposure in systemic circulation in these trials^75–78^. The proportion of patients with anti-drug antibodies for etrolizumab ranged from 18% to 35%, which is higher than was previously observed in phase I and II trials (~5%); however, there was no apparent correlation between anti-drug antibodies and pharmacokinetic parameters^75–78^. One potential explanation is that inhibition of αEβ7 integrin and E-cadherin binding may result in loss of IEL and Trm cell populations, which may have a protective role in IBD. As described previously, IELs defend against infection and maintain the integrity of the intestinal epithelium by preventing uncontrolled immune cell infiltration and excessive immune responses mediated by T cells, although their role in IBD has not been fully elucidated^35^. Additionally, patients with endoscopically active IBD have been shown to have lower percentages of αE+ T cells, including both CD4+ and CD8+ subsets, than healthy controls and patients with IBD who are in endoscopic remission^113,114^. Furthermore, in a phase II study of etrolizumab in patients with UC, a numerically greater proportion of patients with high baseline colonic αE expression levels and high numbers of αE+ cells achieved clinical remission than those with low expression and low numbers of αE+ cells^107^.

As expression of αEβ7 integrin and E-cadherin is not gut-selective, etrolizumab may feasibly affect diverse immune cell subsets expressing αEβ7 integrin in the peripheral blood and other epithelial compartments^108^. Although such interactions could have potential implications for safety, the incidences of serious adverse events and serious infections were similar for etrolizumab and placebo in the phase III trials in UC except for HICKORY, in which a slightly greater proportion of patients who received etrolizumab experienced a serious adverse event than those who received placebo during the maintenance phase^75–77^. In GARDENIA, the incidences of serious adverse events and serious infections were higher for the etrolizumab group than for the infliximab group^78^. Notably, there were no incidences of PML in any of the phase III trials.

Vedolizumab is a humanized mAb that specifically targets the α4β7 integrin heterodimer^81^. The intravenous formulation of vedolizumab is approved for induction and maintenance treatment in Europe, the USA, and Japan, while the subcutaneous formulation is approved for maintenance treatment in Europe^81,82,115,116^. In contrast to etrolizumab and natalizumab, which also inhibit interactions between other integrin heterodimers and their receptors, vedolizumab only inhibits the binding between α4β7 integrin and MAdCAM-1, which mediates the gut-selective trafficking of immune cells. The gut-selective MoA of vedolizumab has been confirmed in an immune challenge study, which demonstrated that the humoral response to a parenterally administered hepatitis B vaccine was similar among individuals who received a single dose of either vedolizumab or placebo, whereas the humoral response to an oral cholera vaccine was significantly reduced in those who received vedolizumab compared with placebo^117^. Furthermore, serologic response to the COVID-19 mRNA vaccine in patients treated with vedolizumab has been shown to be similar to that of healthy individuals, whereas serologic responses were significantly lower in patients receiving an anti-TNFα treatment (infliximab or adalimumab)^118^.

Vedolizumab can bind to several immune cell types that express α4β7 integrin, including a subset of CD4+ memory T cells, eosinophils, naïve T helper cells, naïve and memory cytotoxic T cells, B cells, natural killer cells, and basophils^119^. In some studies, vedolizumab has been shown to inhibit the trafficking of CD4+ and CD8+ T cells to the intestinal mucosa in patients with IBD^120,121^; however, other studies have observed no change in the relative abundance of lamina propria CD4+ and CD8+ T cells after vedolizumab treatment^122,123^. There is also conflicting evidence regarding the impact of vedolizumab on the trafficking of Treg cells^122,124^. In 2022, a study demonstrated that higher concentrations of vedolizumab are necessary to block α4β7 integrin interactions on Treg cells than on Teff cells, which suggests that there is an optimal therapeutic window during which vedolizumab blocks Teff cell trafficking while permitting the residual homing of Treg cells^125^.

Inhibiting the trafficking of naïve T and B cells to intestinal inductive sites and/or minimizing their activation may contribute to the MoA of vedolizumab^123,126^. Naïve T and B cells traffic to inductive sites via α4β7 integrin and MAdCAM-1 binding^25,126^, and evidence from *in vitro* studies also suggests that MAdCAM-1 can costimulate naïve T-cell proliferation via ligation with α4β7 integrin^127,128^. In a small cohort of patients with mild IBD and concomitant HIV-1 infection, vedolizumab reduced levels of B cell subsets and naïve T cells and substantially decreased the size and number of lymphoid aggregates in the terminal ileum^126^. These findings have been substantiated in a larger cohort of patients with UC (*n* = 83), with significant reductions observed in the frequency of naïve B and T cells in vedolizumab-treated patients compared with anti-TNFα-treated and untreated patients^123^. Therapeutic response to vedolizumab was accompanied with a significant attenuation of lymphoid aggregates in a separate cohort of 34 patients with UC^123^. Additionally, lower levels of naïve lymphocytes and myeloid dendritic cells have been observed in mucosal biopsies from patients treated with vedolizumab than in patients who received a different IBD medication^129^. Collectively, these data support the hypothesis that, in addition to inhibiting the trafficking of the effector immune populations, vedolizumab may impact the sites of induction of immune responses. Vedolizumab treatment has also been shown to affect the relative abundance of M1 and M2 macrophages^122^; however, this is likely a secondary effect of attenuated inflammation^121^ because precursors of these cells predominantly traffic via interactions between αLβ2 and αMβ2 integrins with ICAM-1^6^.

In the phase III GEMINI 1 (*n* = 895), GEMINI 2 (*n* = 1115), and GEMINI 3 (*n* = 416) clinical trials, vedolizumab demonstrated superior efficacy to placebo for the induction and maintenance of remission in patients with moderately to severely active UC or CD^130–132^. Furthermore, in the VARSITY trial (*n* = 769), vedolizumab was superior to adalimumab for achievement of clinical remission in patients with moderate to severe UC^133^. Vedolizumab has also demonstrated efficacy over placebo for chronic pouchitis after ileal pouch–anal anastomosis in patients with UC in the EARNEST trial (*n* = 102)^134^.

The presence of anti-drug antibodies does not appear to be associated with vedolizumab treatment response^135^. However, low serum concentrations of vedolizumab have been proposed as a potential explanation for lack of response in some patients, leading some to argue for therapeutic drug monitoring to reach a minimum trough-level serum concentration^125^. Supporting this hypothesis, post hoc analysis of data from GEMINI demonstrates that higher trough concentrations of vedolizumab are associated with higher rates of clinical remission^136,137^. However, patients with more severe disease at the initiation of treatment have lower vedolizumab levels during treatment and are less likely to reach therapeutic targets^138^. Furthermore, clinical doses of vedolizumab have been shown to result in complete occupancy of α4β7 integrin on peripheral blood T and B cells and intestinal CD4+ T cells regardless of response or nonresponse^121,135^. Conversely, very high serum concentrations of vedolizumab block the trafficking of anti-inflammatory Treg cells and may be associated with worse outcomes^125^. ENTERPRET (*n* = 278) was a phase IV trial that aimed to investigate whether optimizing vedolizumab dosage based on serum vedolizumab levels improved clinical outcomes in patients with high drug clearance who did not respond to vedolizumab at week 6. At week 30, patients who received dose-optimized vedolizumab had similar rates of endoscopic mucosal healing to those who received the standard vedolizumab dose, suggesting a limited value for dose optimization in this patient cohort^139^.

Phase III clinical trials and a subsequent post-marketing safety study have established the safety profile of vedolizumab as one that is consistent with its gut-selective MoA^130–132,140^. In a long-term extension of the GEMINI trials, the rate of serious infection was 18.0 cases per 1000 patient-years for UC and 33.6 cases per 1000 patient-years for CD^140^. Only one case of PML has been reported in a patient receiving vedolizumab; however, an independent adjudication committee determined that the probable cause was the patient’s HIV+ status and their long-term use of immunosuppressant medication^141^. Furthermore, evidence suggests that vedolizumab is associated with a lower risk of infections or serious infections than anti-TNFα treatments^133,142^. Although it has been postulated that, owing to its gut-selective MoA, vedolizumab could increase susceptibility to gastrointestinal infections specifically, a retrospective analysis of real-world data found that vedolizumab was associated with a lower risk of *Clostridioides difficile* infection than anti-TNFα treatments in patients with UC^143^.

The efficacy and safety profile of vedolizumab has generated interest in the development of new α4β7 integrin antagonists, including MORF-057, an orally administered small molecule antagonist^83^, and abrilumab, a human monoclonal IgG_2_ antibody, which are in early clinical development phases^84^. Orally administered peptide antagonists are also being investigated. One such peptide, PTG-100, demonstrated target engagement and was well tolerated after single and multiple dose administration in a phase I first-in-human trial in healthy volunteers. However, the subsequent phase IIa trial in patients with moderately to severely active UC was prematurely terminated owing to lack of efficacy following an interim analysis^144^. PN-10943, a second-generation peptide antagonist that is more potent than PTG-100, is now being clinically developed^85^.

### Anti-adhesion molecule therapies

Alicaforsen is an antisense oligonucleotide therapy that targets ICAM-1^86^. Interactions between β2-integrins and ICAM-1 mediate the adhesion of immune cells to the vascular endothelium and facilitate their transmigration to the intestinal mucosa^145^. In two early clinical trials (*n* = 331; *n* = 299), intravenously administered alicaforsen failed to demonstrate efficacy over placebo in patients with active CD^86,87^. When administered as an enema formulation in patients with mild to moderate left-sided UC (*n* = 112; *n* = 190), alicaforsen did not meet its primary endpoint at week 6 versus placebo or mesalazine, but reductions in disease activity index from baseline were observed at week 18 in patients who received alicaforsen^88,89^. These fairly promising results when administered as an enema formulation may be because anti-adhesion molecule therapies need to be delivered to the vascular endothelium in order to interact with ICAM-1^145^. Alicaforsen has also been investigated in a phase III clinical trial (*n* = 138) for the treatment of chronic pouchtitis as an enema formulation. No statistically significant differences in modified Mayo Endoscopy Score were observed between alicaforsen and placebo; however, the authors concluded that this measure was inappropriate for patient selection and disease assessment in chronic pouchtitis^90^.

Ontamalimab is a subcutaneously administered human mAb targeting MAdCAM-1, which inhibits the interaction between α4β7 integrin and MAdCAM-1 necessary for gut-selective trafficking and therefore acts via a similar mechanism as established anti-integrin therapies for IBD^91,92^. In the phase II TURANDOT trial (*n* = 357), remission rates were significantly greater for ontamalimab than placebo in patients with moderately to severely active UC^91^; whereas in the OPERA study (*n* = 265), clinical response was similar for ontamalimab and placebo in patients with moderately to severely active CD^92^. The clinical development program for ontamalimab has since been discontinued^93^.

### S1P receptor modulators

Ozanimod is an orally administered small molecule agonist of S1P_1_ and S1P_5_ receptors that has been approved in Europe and the USA for the treatment of moderately to severely active UC^96,97^. S1P_1_ agonists overstimulate S1P_1_ receptors, promoting receptor internalization and thereby acting as functional antagonists. Downregulation of the S1P_1_ receptor on lymphocytes inhibits their ability to respond to the S1P gradient and egress from peripheral lymphoid tissue, resulting in their sequestration^146^. Sequestered lymphocytes cannot enter the bloodstream and traffic to sites of inflammation, such as the gut^94^. In the phase III True North trial (induction, *n* = 1012; maintenance, *n* = 457), ozanimod was superior to placebo for the induction and maintenance of clinical remission in patients with moderately to severely active UC^95^. Initiation of ozanimod may also result in a transient decrease in heart rate and atrioventricular conduction delays^96^, which is likely due to agonism of S1P_1_ receptors on cardiac myocytes^146^. Partial agonist activity for other S1P receptor sub-types (S1P_2–5_) may have further potential implications for the safety of S1P receptor modulators^94,96,98,100,101,147^.

Several other oral small molecule S1P receptor modulators are in clinical development for IBD, including: etrasimod, an agonist of S1P_1_ receptors and a partial agonist of S1P_4_ and S1P_5_ receptors^98^; mocravimod, an agonist of the S1P_1_ receptor and a partial agonist of the S1P_3_ receptor^100^; and amiselimod, an agonist of S1P_1_ and S1P_5_ receptors^101^. Etrasimod met all primary and secondary efficacy endpoints versus placebo in patients with moderately to severely active UC in the phase III ELEVATE UC 12 (*n* = 354) and ELEVATE UC 52 (*n* = 433) trials^99^. In contrast, mocravimod failed to meet its primary efficacy endpoint in a phase II proof-of-concept trial (*n* = 27) in patients with moderately active UC and an inadequate response to 5-aminosalicylate therapy^100^. Additionally, in a proof-of-concept study (*n* = 78), no improvements in clinical or biochemical disease activity were observed with amiselimod compared with placebo in patients with treatment-refractory active CD^102^.

## EVOLUTION OF MOLECULAR RESISTANCE

Patients with IBD may not respond to treatment (primary nonresponse) or may respond initially but subsequently lose response during maintenance therapy (secondary loss of response)^148^. Secondary loss of response to anti-TNFα treatment is commonly caused by low trough serum drug levels due to immunogenicity, nonadherence, fecal drug loss, or nonimmune clearance^148^. Low serum drug levels due to the presence of anti-drug antibodies have also been postulated as a potential reason for nonresponse to other biologics^149^.

Alternatively, nonresponse may arise from the development of molecular resistance^150^. A mechanism for the evolution of molecular resistance has been proposed in which the treatment exerts a selection pressure in the intestinal mucosa. Immune cells that are sensitive to the treatment undergo apoptosis, whereas resistant immune cells expand and become the dominant immune cell phenotype. The composition of the immune cells in the intestinal mucosa therefore changes over time with treatment, which can impact response to subsequent therapies^150^. Most data on the development of molecular resistance pertain to anti-TNFα treatment and are associated with the activation of alternative cytokine pathways^150–154^.

Theoretically, there may be less potential for molecular resistance to develop for anti-trafficking therapies than anticy-tokines because the former prevent immune cells from trafficking to the inflammatory environment and thus prevent their exposure to the selection pressures that drive the development of molecular resistance. In contrast, anti-cytokines do not provide separation of immune cells from the inflammatory environment, in which redundant inflammatory pathways may compromise response. Because the development of molecular resistance changes the composition of immune cells in the intestinal mucosa, it may also affect response to subsequent treatments, highlighting the importance of both the choice of treatment and the sequence of therapies^150^. For example, vedolizumab appears to have greater efficacy in patients who are naïve to biologic therapies than in those who have previously been treated with an anti-TNFα agent^155,156^, whereas failure of vedolizumab as a first-line biologic does not appear to influence subsequent response to anti-TNFα treatments^142,157^. Further research on other anti-trafficking and anti-cytokine therapies is needed, however, to investigate the impact of treatment sequence on efficacy.

## COMBINATION THERAPY

In recent years, the use of combination therapy, in which two or more advanced therapies are prescribed concurrently, has been considered for IBD, particularly for patients with refractory uncontrolled IBD or those with well-controlled IBD but with uncontrolled extraintestinal symptoms^158,159^. Except for one randomized controlled trial investigating infliximab plus natalizumab versus infliximab monotherapy published in 2007^160^, most data have been limited to case reports or case series until recently^158,159^. A systematic review of the literature conducted in 2020 identified 279 patients with IBD treated with dual advanced therapy (dual biologic or a biologic plus tofacitinib). Evidence for the effectiveness of combination therapy is promising, with pooled rates of clinical and endoscopic remission of 58.8% and 34.3%, respectively, in these patients, despite the majority having treatment-refractory disease^161^.

In addition to inducing remission in treatment-refractory patients, combination therapy could improve the efficacy achieved with existing monotherapies and target new endpoints such as disease clearance, which includes clinical, endoscopic, and histologic remission in UC as well as potentially transmural healing in CD^162^. However, combining two immunosuppressive therapies could increase the risk of adverse events, such as serious infection and malignancy^163^. The MoA of drugs included in any potential combination therapy is particularly relevant to this discussion because a systemically acting therapy plus a gut-selective therapy would be expected to have less of a systemic immunosuppressive effect than two systemically acting therapies combined. Vedolizumab is commonly included in combination therapies, likely owing to its gut-selective MoA and established safety profile. In one of the larger retrospective cohort studies on dual biologic or advanced therapy (*n* = 50), vedolizumab plus ustekinumab (67.6%) was the most common combination therapy observed for patients with CD, whereas vedolizumab plus tofacitinib (44.4%) was the most common combination therapy for patients with UC^163^. With regard to safety, pooled rates of adverse events, infections, and malignancy for combination therapy across studies are reportedly similar to those observed with anti-TNFα monotherapy^161^. However, further research is needed because the safety profile will likely differ among different combinations. Some combinations also include a conventional therapy, such as an immunomodulator, which may further increase the risk of serious infections^163^.

Although interest in combination therapy is growing, few prospective interventional studies are being conducted to investigate the efficacy and safety of potential treatment combinations^164,165^. VEGA is a recently completed phase II clinical trial that investigated the efficacy and safety of combination therapy with two anti-cytokines, golimumab and guselkumab, in patients with UC^166^. At week 12, 83% of patients who received combination therapy (*n* = 71) had a clinical response, compared with 61% and 75% who received golimumab monotherapy (*n* = 72) and guselkumab monotherapy (*n* = 71)^166^. EXPLORER is an open-label phase IV trial investigating the effect of triple combination therapy with vedolizumab, adalimumab, and methotrexate in patients newly diagnosed with high-risk CD^164^. In a prespecified interim analysis, 34.5% and 54.5% of patients (*n* = 55) were in endoscopic and clinical remission, respectively, after 26 weeks of triple combination therapy, with no new safety signals observed^167^. For context, the reported rate of endoscopic remission at week 26 was 19.6% in anti-TNFα treatment naïve patients with CD receiving vedolizumab (*n* = 46) in the VERSIFY study^168^ and 28% at week 52 in patients with CD treated with continuous adalimumab (*n* = 64) in the Extend the Safety and Efficacy of Adalimumab through Endoscopic Healing (EXTEND) study^169^, which suggests a relative benefit of combination therapy. However, in the LOw Countries VEdolizumab in CD (LOVE-CD) trial, 33% of patients with CD (*n* = 110) receiving vedolizumab monotherapy achieved endoscopic remission at week 26^170^. Further studies are therefore needed to confirm any potential benefit.

## CONCLUSION

The controlled trafficking of immune cells into the intestinal mucosa plays an essential role in gut homeostasis and immunosurveillance. In IBD, aberrant trafficking of immune cells results in an accumulation of T cells that perpetuates the inflammatory immune response. Whereas anti-cytokines neutralize either one or two cytokines or inhibit cytokine signaling pathways within the gut, anti-trafficking therapies limit the trafficking of immune cells to the gut. Anti-trafficking therapies act via a range of different mechanisms, with implications for their efficacy and safety. Although evidence is currently limited, anti-trafficking therapies may be associated with less risk of developing molecular resistance than anti-cytokines, which has implications for treatment sequencing. Furthermore, the mechanisms of action of advanced therapies should be an important consideration for combination therapy, given the potential risk of serious infections arising from use of multiple immunosuppressive drugs.

## Figures and Tables

**Table 1.** Summary of anti-trafficking therapies investigated for the treatment of inflammatory bowel disease.

Drug name	Type	Target(s)	Clinical development or approval status	Approved dosing regimen
Vercirnon	Small molecule antagonist^63^	CCR9^63,64^	Clinical development terminated^64^	N/A

Eldelumab	Fully human monoclonal antibody^65–67^	CXCL10^65–67^	No clinical development plans reported	N/A
GSK3050002	Humanized monoclonal antibody^68,69^	CCL20^68,69^	Clinical development terminated^69^	N/A
Natalizumab	Humanized monoclonal antibody^70^	α4 subunit of α4β7 and α4β1 integrins^71^	Approved by the FDA for the treatment of adult patients with moderately to severely active CD with evidence of inflammation who have had an inadequate response to, or are unable to tolerate, conventional CD therapies and inhibitors of TNFα^72^	300 mg IV Q4W^72^
Carotegrast methyl	Small molecule antagonist^73^	α4 subunit of α4β7 and α4β1 integrins^73^	Approved by the PMDA for the treatment of moderate ulcerative colitis (for use only in patients who have not sufficiently responded to 5-aminosalicyclic acid preparation)^74^	N/A
Etrolizumab	Humanized monoclonal antibody^75–78^	β7 subunit of α4β7 and αEβ7 integrins^75–79^	Clinical development terminated^80^	N/A
Vedolizumab	Humanized monoclonal antibody^81^	α4β7 integrin^81^	Approved by the EMA, FDA, and PMDA for the treatment of adult patients with moderately to severely active UC or CD^81–116,a^	300 mg IV at 0, 2, and 6 weeks, then Q8W thereafter^81,82^ EMA: Following at least 2 VDZ IV infusions, VDZ SC 108 mg Q2W thereafter^82^
MORF-057	Small molecule antagonist^83^	α4β7 integrin^83^	In clinical development^83^	N/A
Abrilumab	Human monoclonal antibody^84^	α4β7 integrin^84^	In clinical development^84^	N/A
PN-10943	Peptide antagonist^85^	α4β7 integrin^85^	In clinical development^85^	N/A
Alicaforsen	Antisense oligonucleotide^86–90^	ICAM-1^86–90^	In clinical development^86–90^	N/A
Ontamalimab	Fully human monoclonal antibody^91,92^	MAdCAM-1^91,92^	Clinical development terminated^93^	N/A
Ozanimod	Small molecule agonist^94^	S1P_1_ and S1P_5_ receptors^94,95^	Approved by the EMA and FDA for the treatment of adult patients with moderately to severely active UC^96,97,b^	0.23 mg OD oral on days 1–4, 0.46 mg OD oral on days 5–7, and 0.92 mg OD on day 8 and thereafter^96,97^
Etrasimod	Small molecule agonist^98,99^	S1P_1_ receptor (partial agonist of S1P_4_ and S1P_5_ receptors)^98,99^	In clinical development^99^	N/A
Mocravimod	Small molecule agonist^100^	S1P_1_ receptor (partial agonist of the S1P_3_ receptor)^100^	In clinical development^100^	N/A
Amiselimod	Small molecule agonist^101^	S1P_1_ and S1P_5_ receptors (minimal agonist of S1P_4_ receptor)^101^	In clinical development^101,102^	N/A

CCL = C-C chemokine ligand; CCR = C-C chemokine receptor; CD = Crohn’s disease; CXCL = C-X-C ligand; EMA = European Medicines Agency; FDA = US Food and Drug Administration; PMDA = Pharmaceuticals and Medical Device Agency Japan; ICAM-1= intracellular adhesion molecule 1; IV = intravenous; MAdCAM-1 = mucosal vascular addressin cell adhesion molecule 1; N/A = not available; OD = once daily; Q4W = every 4 weeks; Q8W = every 8 weeks; S1P = sphingosine-1 phosphate; TNF = tumor necrosis factor; UC = ulcerative colitis.

a EMA specifies for use in patients who have had an inadequate response with, lost response to, or were intolerant to either conventional therapy or a TNFα antagonist, whereas the PDMA specifies for use only in patients who have not sufficiently responded to conventional treatments.

b EMA specifies for use in patients who have had an inadequate response, lost response, or were intolerant to either conventional therapy or a biologic agent.
